# p38 ^MAPK^ stress signalling in replicative senescence in fibroblasts from progeroid and genomic instability syndromes

**DOI:** 10.1007/s10522-012-9407-2

**Published:** 2012-10-31

**Authors:** Hannah S. E. Tivey, Amy J. C. Brook, Michal J. Rokicki, David Kipling, Terence Davis

**Affiliations:** Institute of Cancer and Genetics, School of Medicine, Cardiff University, Heath Park, Cardiff, CF14 4XN UK

**Keywords:** F-actin stress fibres, SB203580, Human telomerase, Cellular morphology, Human ageing, p38 MAP kinase inhibitors, Werner syndrome

## Abstract

Werner Syndrome (WS) is a human segmental progeria resulting from mutations in a DNA helicase. WS fibroblasts have a shortened replicative capacity, an aged appearance, and activated p38 MAPK, features that can be modulated by inhibition of the p38 pathway. Loss of the WRNp RecQ helicase has been shown to result in replicative stress, suggesting that a link between faulty DNA repair and stress-induced premature cellular senescence may lead to premature ageing in WS. Other progeroid syndromes that share overlapping pathophysiological features with WS also show defects in DNA processing, raising the possibility that faulty DNA repair, leading to replicative stress and premature cellular senescence, might be a more widespread feature of premature ageing syndromes. We therefore analysed replicative capacity, cellular morphology and p38 activation, and the effects of p38 inhibition, in fibroblasts from a range of progeroid syndromes. In general, populations of young fibroblasts from non-WS progeroid syndromes do not have a high level of cells with an enlarged morphology and F-actin stress fibres, unlike young WS cells, although this varies between strains. p38 activation and phosphorylated HSP27 levels generally correlate well with cellular morphology, and treatment with the p38 inhibitor SB203580 effects cellular morphology only in strains with enlarged cells and phosphorylated HSP27. For some syndromes fibroblast replicative capacity was within the normal range, whereas for others it was significantly shorter (e.g. HGPS and DKC). However, although in most cases SB203580 extended replicative capacity, with the exception of WS and DKC the magnitude of the effect was not significantly different from normal dermal fibroblasts. This suggests that stress-induced premature cellular senescence via p38 activation is restricted to a small subset of progeroid syndromes.

**Electronic supplementary material:**

The online version of this article (doi:10.1007/s10522-012-9407-2) contains supplementary material, which is available to authorized users.

## Introduction

Much remains to be discovered regarding the pathophysiology of human senescence (Puzianowska-Kuznicka and Kuznicki 2005), a complex process involving genetic and environmental factors affecting several physiological pathways. One mechanism commonly postulated as underlying human ageing is replicative cellular senescence, or the observation that many normal human somatic cells are capable of only a finite number of divisions (Campisi 1996; Ostler et al. 2002; Burton 2009; Faragher et al. 2009). Replicative senescence in fibroblasts results from the progressive loss of telomeric repeats at the ends of chromosomes due to the inability of the DNA replication machinery to efficiently duplicate the 5′ ends of linear chromosomes (Harley et al. 1990; Hastie et al. 1990; Blackburn 1991; Allsopp et al. 1992; Vaziri et al. 1994). Telomeres shorten with each cell generation until a critical length is reached whereupon they lose their function, resulting in a signal to exit the cell cycle (d’Adda di Fagagna et al. 2003).

Replicative senescence can contribute to age-related degenerations in division competent tissues as a result of reduced proliferative capacity in organs where cell division is central to normal function or repair (e.g. small intestine, immune system, skin), or from the observation that senescent cells display deleterious biochemical features as a result of patterns of gene expression that differ markedly from their dividing counterparts (Ostler et al. 2002; Kipling et al. 2004; Burton 2009; Faragher et al. 2009). For example, senescent cells secrete inflammatory cytokines such as IL-1 and tumour necrosis factor (TNFα) (Kumar et al. 1993; Parrinello et al. 2005), and express cell surface molecules such as ICAM-1 that are involved in the recruitment of leukocytes during inflammation (Gorgoulis et al. 2005).

Evidence for replicative senescence in vivo has been difficult to acquire, and a possible relationship between the replicative capacity of fibroblasts in vitro and human age has been found lacking in a re-evaluation (Cristofalo et al. 1998), leading to criticism of this postulate (Rubin 2002). However, several observations suggest that senescent cells do occur in vivo and accumulate with age (Lindsey et al. 1991; Vaziri et al. 1994; Chang and Harley 1995; Dimri et al. 1995; Paradis et al. 2001; Vasile et al. 2001; Minamino et al. 2002; Herbig et al. 2006; Jeyapalan et al. 2007), and modulations that lengthen life span, such as dietary restriction in mice, reduce the rate of accumulation of senescent cells in the eye (Li et al. 1997).

Various practical difficulties underlie human ageing studies, most importantly the polygenic nature of many of the associated pathologies. An alternative to the study of whole body ageing in normal humans is the study of progeroid syndromes whose phenotypes show specific characteristics of ageing (Hofer et al. 2005; Kudlow et al. 2007). Most progeroid syndromes where the aetiological factors are known are monogenic and segmental, in that they show many, but not all, of the clinical characteristics of normal ageing. In those aspects where premature ageing occurs, the process and pathology are remarkably similar to that seen in normally aged individuals (Hofer et al. 2005; Kudlow et al. 2007).

One of the more intensively studied progeroid syndromes is Werner (WS), with affected individuals showing premature onset of cataracts, skin atrophy, hair-greying and soft tissue calcification, together with age-related diseases such as type II diabetes, atherosclerosis and osteoporosis (Martin et al. 1999). Associated with the premature skin ageing, accelerated cellular replicative senescence is found in WS fibroblasts (Tollefsbol and Cohen 1984). This accelerated senescence of WS cells in vitro has been postulated to correspond to a similar process in vivo, and thus contribute to the accelerated ageing of division-competent tissues (Ostler et al. 2002).

Likewise, many strains of Hutchinson-Gilford Progeria (HGPS) fibroblasts show reduced replicative capacity, although increased apoptosis is also prevalent, and HGPS individuals undergo rapid ageing and have very short lifespans (Brown 1992; Hofer et al. 2005). With Ataxia-telangiectasia (AT), the replicative capacity of fibroblasts is significantly reduced compared to normal (Elmore and Swift 1976; Tchirkov and Lansdorp 2003). Individuals with AT show moderate features reminiscent of premature ageing, such as grey hair, wrinkled skin, skin atrophy and sclerosis, and show a reduced lifespan with death usually occurring in the third and fourth decades (Taylor et al. 1996).

In addition to replicative senescence, human fibroblasts can undergo stress-induced premature senescence (SIPS) via activation of the MAP kinase p38 that responds to and is activated by endogenous and exogenous cellular stress (Freund et al. 2011). p38 is involved in growth arrest in response to the expression of oncogenes such as *ras* (Wang et al. 2002; Deng et al. 2004), exogenous stress such as arsenite treatment or oxidative stress (Guay et al. 1997) and in telomere-dependent senescence (Iwasa et al. 2003): indeed, p38 defines a common senescence signalling pathway (Iwasa et al. 2003). p38 is important for senescence growth arrest due to its ability to activate both the p53/p21^WAF1^ and pRb/p16^INK4A^ growth arrest pathways.

Recent work using young WS fibroblasts has shown that both the cellular replicative capacity and aged morphology of WS fibroblasts can be reverted to that seen for normal fibroblasts by treatment with the p38 inhibitor SB203580 (Davis et al. 2005; Davis et al. 2006). This, together with the observation of active p38 stress signalling, suggests that a form of SIPS is active in WS cells. As replicative senescence in WS cells is telomere dependent (Davis et al. 2003), this suggests that SIPS acts in synergy with telomere-shortening to result in the premature cellular replicative capacity (Davis and Kipling 2006). WS is associated with inflammatory conditions, such as atherosclerosis, diabetes and osteoporosis (Murano et al. 1997; Choi et al. 2001; Yokote et al. 2004; Davis and Kipling 2006). Because p38 activation leads to the production of pro-inflammatory cytokines such as IL-1 and TNFα (Ferran et al. 1995), it is possible that elevated p38 activity in vivo in WS individuals may lead to the observed elevated levels of p38-inducible inflammatory cytokines, and thus plays a role in WS pathophysiology (Davis and Kipling 2006).

In contrast, although AT fibroblasts have a shorter replicative capacity than normal fibroblasts, they do not show an aged-morphology or have activated p38, and inhibition of p38 does not extend their replicative capacity beyond that seen in normal fibroblasts (Davis and Kipling 2009). In addition, AT individuals do not show inflammatory features (Wood et al. 2001; Barzilai et al. 2002; Hofer et al. 2005). This suggests that SIPS is not a feature of AT, and that the shortened replicative capacity of AT fibroblasts results from a different process, such as accelerated telomere shortening (Tchirkov and Lansdorp 2003).

Based upon these studies it has been suggested that premature cellular senescence and p38 activation may underlie many of the premature ageing features of other of these progeroid syndromes (Davis and Kipling 2006). To address this question requires a greater understanding of the replicative capacity and p38 stress-signalling pathway in these syndromes. We therefore sought to determine whether the lesions present in these syndromes result in p38 activation and a phenotype similar to SIPS as a result of endogenous physiological levels of stress (which is distinct from their ability to respond to exogenously applied stress), and thus whether a SIPS-like response is a common response in progeroid syndromes. We have therefore determined the growth characteristics and replicative capability of fibroblasts from several progeroid syndromes and investigated the role played by p38 MAP kinase, using a combination of molecular profiling and small molecule inhibitor use. Furthermore, because telomere shortening is known to synergise with SIPS in WS fibroblasts and is a major mechanism driving fibroblast senescence, we have also used ectopic expression of human telomerase to determine whether replicative senescence in fibroblasts from progeroid syndromes is telomere-dependent.

## Materials and methods

### Cell culture

Primary dermal fibroblasts for the majority of the syndromes used in this work were derived from biopsies of human tissue and obtained from the Coriell Cell Repository (Camden, NJ, USA). The two NBS strains RO202 and RO242 were obtained from W.J. Kleijer. For clarity, when referring to cell strains each strain code is represented by an abbreviated syndrome prefix with the strain code in brackets, e.g., SS(GM09812) for *S*eckel *S*yndrome cells (see Supplementary Table 1). Normal dermal fibroblasts (NDFs) are given the prefix N. HCA2^tert^ cells have been described previously (Davis and Kipling 2009). The genetic lesions found in the cell strains used in this study are shown in supplementary Table 2, as far as they are known.

All cells were grown in Earle’s modified Eagle medium (EMEM: Gibco Invitrogen, Paisley, UK) supplemented with 10 % foetal calf serum (Autogen Bioclear, Salisbury, UK), 1× vitamins, 2 mM l-glutamine (Invitrogen Life Technologies Ltd., Paisley, UK), 1× essential amino acids, 1× non-essential amino acids, 1 mM sodium pyruvate, 10,000 U/ml penicillin and 10 mg/ml streptomycin (Sigma, Poole, UK) in an atmosphere of 21 % O_2_ and 5 % CO_2_, and passaged every 4–5 days as described previously (Davis et al. 2003). Cultures were not allowed to become confluent at any time so as to maintain maximal growth rates. Population doublings (PDs) were calculated according to the formula: PDs = log(*N*_t_/*N*_o_)/log2, where *N*_t_ is number of cells counted and *N*_o_ is number of cells seeded. The initial growth rates for all cultures were determined using the first 30 days of culture when the growth was still on the linear part of the growth curve. An example growth curve for each progeroid syndrome is shown in Supplementary Figure 1.

For experiments using p38 MAP kinase inhibitors, the medium was supplemented with 2.5 μM SB203580 (Tocris Chemical Co. Bristol, UK) dissolved in DMSO, with the medium changed daily. For controls, an equivalent volume of DMSO was added to the medium. 2.5 μM was chosen because this concentration is in the range used routinely for studying its effect on p38 activity in cellular systems (Haq et al. 2002; Wang et al. 2002; Iwasa et al. 2003; Davis et al. 2005). We have previously shown that this concentration effectively inhibits p38 while not significantly inhibiting the related JNK1/2 kinases (Bagley et al. 2010).

To activate the p38-signalling pathway using anisomycin, N(AG16409)^tert^ or HCA2^tert^ cells were plated onto 100 mm dishes in EMEM and cultured for 2 days at 37 °C, after which the cells were treated with 30 μM anisomycin (Sigma, Poole, UK) for 45 min.

### Immunofluorescence microscopy

Actin staining for immunofluorescence microscopy was performed essentially as described (Huot et al. 1997). Briefly, the cells were plated into 35 mm plastic dishes in EMEM and allowed to settle for 48 h. The cells were then washed in PBS, fixed in 3.7 % (w/v) paraformaldehyde for 20 min and permeabilised with 0.1 % (v/v) Triton-X100 for 20 min. F-actin was detected using fluorescein isothiocyanate-conjugated (FITC)-conjugated phalloidin (33 μg/ml) (Sigma, Poole, UK), diluted 1:50 in PBS for 30 min in the dark, followed by washing in PBS.

### Immunoblot analysis

Protein samples were prepared in lysis buffer containing the phosphatase inhibitors NaF and Na_3_VO_4_, separated on 12 % (w/v) sodium-dodecylsulphate/polyacrylamide electrophoresis gels, electroblotted to Immobilon-P polyvinylidene difluoride or nitrocellulose membranes (Millipore, Watford, UK) and antibodies applied as described previously (Davis et al. 2005). The antibodies used were: mouse monoclonal anti-HSP27 (G31), rabbit polyclonal anti-phospho(S82)-HSP27, anti-p38 and anti-phospho(T180/Y182)-p38 (Cell Signalling, New England Biolabs, Hitchin, UK). An enhanced chemiluminescence kit (GE Healthcare, Chalfont St Giles, UK) was used for visualization using HRP-coupled goat secondary antibodies.

### Retroviral gene transfer

pBABE-hTERT is an amphotropic retrovirus expressing the catalytic protein subunit of human telomerase hTERT (Wyllie et al. 2000). For control infections pBABE-puro vectors packaged in ψCRIP cells were used. Gene transfer was carried out as described previously (Davis et al. 2003). 100,000 cells were plated onto 100 mm culture dishes in EMEM and left to settle for 48 h at 37 °C, after which time polybrene was added to a final concentration of 8 μg/ml for 1 h. The medium was then replaced with 5 ml of viral supernatant in growth medium (either pBABE-hTERT-puro or pBABE-puro at approximately 1 × 10^5^ viral particles per ml) and the cells incubated at 37 °C for 24 h. For each gene transfer the retroviral infections were performed twice. 24 h after the second infection, fibroblast cultures were passaged into EMEM containing puromycin at 2.5 μg/ml and surviving cells after 5 days were transferred to T75 culture vessels and cultured in EMEM containing puromycin and grown until they had surpassed at least twice the replicative capacity of the uninfected controls. Cell strains expressing telomerase activity are given the suffix ‘tert’.

### Detection of telomerase activity

Telomerase was assayed in whole cell extracts using the TRAP assay as described (Kim and Wu 1997). The cell line 293 provided a telomerase-positive control. Reaction products were separated on non-denaturing 10 % polyacrylamide gels and visualized by Sybr Gold staining and fluorimaging on a STORM phosphorimager using blue fluorescence mode (AP Biotech).

## Results

### Effects of the p38 MAPK inhibitor SB203580 on the growth of normal human fibroblasts

**Fig. 1:**
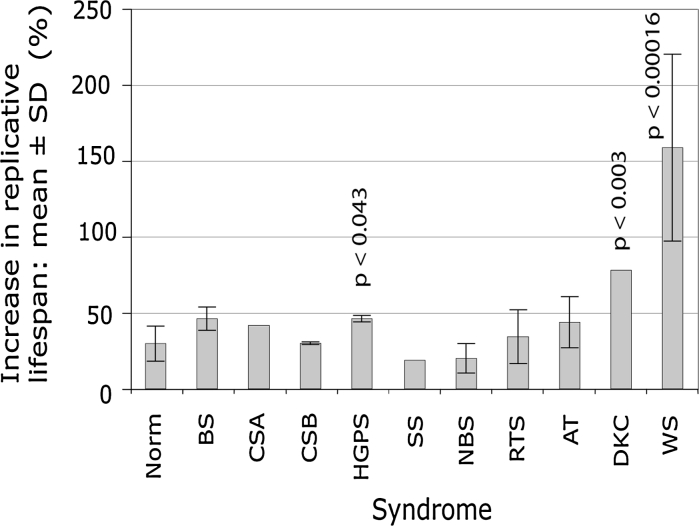
*Bar chart* showing mean percentage increase in experimental replicative capacity using SB203580 for each syndrome; the probability that the increase seen is within the range for normal cells is given for those that are statistically significant

In our previous work (Davis and Kipling 2009) we grew dermal fibroblasts (NDF) from four normal individuals in the presence or absence of SB203580 until each strain reached replicative senescence, and in this current study we analysed a further four NDF strains (data summarised in Table 1). NDFs derived from these eight individuals had an average replicative capacity of 38.8 ± 10.5 PDs. This was extended to 46.6 ± 12.1 PDs in the presence of SB202580. This increase in experimental replicative capacity for these normal strains is 29.7 ± 11.6 % (Table 1; Fig. 1). This compares to a replicative capacity extension seen in WS and AT fibroblasts of 158.7 ± 61 % (*p* < 0.00016) and 43.8 ± 16.8 % (*p* > 0.14) respectively (see Fig. 1). The eight strains of NDFs had average initial growth rates of 0.29 ± 0.09 PDs/day, and SB203580 treatment increased the initial growth rate to an average of 0.40 ± 0.13 PDs/day (Table 1).

**Table 1:** Replicative capacity and growth rates of fibroblasts grown in presence or absence of SB203580

Strain	PDs achieved (control)^a^	PDs achieved (SB203580)^a^	% increase in replicative capacity^b^	Growth rate (control)^c^	Growth rate (SB203580)^c^
N(AG04552)	24.3	30.1	40.6	0.17	0.24
N(AG06234)	34.1	41.6	39.3	0.26	0.40
N(AG09603)	47.8	55.7	24.8	0.33	0.40
N(AG11020)	41.7	46.5	14.7	0.39	0.45
N(AG11081)	33.4	39.3	28.9	0.33	0.41
N(AG13152)	28.0	35.6	34.5	0.15	0.19
N(AG13156)	46.6	63.4	42.4	0.36	0.55
N(AG16409)	54.3	60.5	12.3	0.39	0.55
Mean	38.8 ± 10.5	46.6 ± 12.1	29.7 ± 11.6	0.29 ± 0.09	0.4 ± 0.13
BS(GM02520)	19.3	29.3	51.8	0.18	0.40
BS(GM02548)	23.8	35.5	49.1	0.37	0.48
BS(GM02932)	24.5	33.7	37.5	0.32	0.43
Mean	22.5 ± 2.8	32.8 ± 3.2	46.1 ± 7.6	0.29 ± 0.1	0.44 ± 0.04
Probability^d^	*p* > 0.063	*p* > 0.09	*p* > 0.052	*p* > 0.91	*p* > 0.64
CSA(GM01856)	21.1	29.9	41.7	0.37	0.49
Probability^e^	*p* < 0.045	*p* > 0.095	*p* > 0.13	*p* > 0.22	*p* > 0.23
CSB(GM10903)	51.4	67.2	30.7	0.54	0.67
CSB(GM10905)	61.2	79.2	29.4	0.64	0.76
Mean	56.3 ± 6.9	73.2 ± 8.5	30.0 ± 0.9	0.59 ± 0.07	0.71 ± 0.06
Probability^d^	*p* > 0.059	*p* < 0.02	*p* > 0.96	*p* < 0.004	*p* < 0.012
HGPS(AG01972)	14.4	20.9	45.1	0.29	0.40
HGPS(AG10677)	3.8	5.5	44.7	0.13	0.17
HGPS(AG11498)	24.8	32.0	48.6	0.38	0.39
Mean	14.3 ± 10.5	19.5 ± 13.3	46.1 ± 2.1	0.27 ± 0.13	0.32 ± 0.13
Probability^d^	*p* < 0.008	*p* < 0.011	*p* < 0.043	*p* > 0.67	*p* > 0.39
SS(GM09812)	32.9	39.1	18.8	0.50	0.62
Probability^e^	*p* > 0.28	*p* > 0.27	*p* > 0.17	*p* < 0.016	*p* < 0.044
NBS(RO202)	43.1	48.8	13.2	0.34	0.38
NBS(RO242)	53.5	26.9	67.9	0.35	0.41
Mean	48.3 ± 7.3	58.4 ± 13.5	20.0 ± 9.7	0.345 ± 0.007	0.39 ± 0.02
Probability^d^	*p* > 0.26	*p* > 0.26	*p* > 0.31	*p* > 0.51	*p* > 0.96
DKC(GM01774)	11.9	16.5	78.0	0.15	0.22
Probability^e^	*p* < 0.006	*p* < 0.007	*p* < 0.003	*p* > 0.076	*p* > 0.086
RTS cells:					
RTS(AG05013)	45.4	58.9	55.5	0.38	0.55
Mean^f^	30.5 ± 15.3	39.6 ± 18.5	37.5 ± 21.8	0.33 ± 0.07	0.5 ± 0.14
Probability^d^	*p* > 0.51	*p* > 0.44	*p* > 0.24	*p* > 0.59	*p* > 0.22
AT cells^g^					
Mean	19.6 ± 7.2	25 ± 8.02	43.9 ± 16.8	0.24 ± 0.11	0.33 ± 0.06
Probability^d^	*p* < 0.018	*p* < 0.021	*p* > 0.14	*p* > 0.26	*p* > 0.43
WS cells^h^					
Mean	19.9 ± 4.4	36.5 ± 10.6	158.7 ± 61.5	0.11 ± 0.04	0.28 ± 0.09
Probability^d^	*p* < 0.017	*p* > 0.24	*p* < 0.00016	*p* < 0.011	*p* > 0.17

^a^Total PDs achieved including any known PDs cells have already done prior to obtaining them from Coriell Repository (Supplementary Table 1)

^b^The % increase is determined with reference to starting PD as far as possible: e.g., for N(AG04552) the replicative capacity increase is (30.1 PDs − 10 PDs)/(24.3 PDs − 10 PDs) = 1.406 or a 40.6 % increase in experimental replicative capacity

^c^Value determined for the growth during the first 30 days

^d^Probability that mean value is similar to mean value for normal cells: two-tailed *t* test. In these tests untreated syndrome cells are compared to untreated normal cells, and SB203580-treated syndrome cells are compared to SB203580-treated normal cells

^e^Probability that value is within the distribution of normal cells: *z*-test

^f^Mean includes data for three other RTS strains, see (Davis et al. 2012) for actual data

^g^See (Davis and Kipling 2009) for actual data

^h^See (Davis et al. 2005) for actual data

### Effects of SB203580 on the growth of fibroblasts from genomic instability and progeroid syndromes

Fibroblasts from eight different genomic instability and progeroid syndromes (CSA/CSB, BS, RTS, HGPS, SS, DKC and NBS) were grown to replicative senescence in the presence or absence of SB203580 and their replicative capacities and growth rates recorded. These were compared to previous data for WS and AT (all growth data are summarised in Table 1). An illustrative growth curve for each of the eight syndromes in used this work syndrome is shown in Supplementary Figure 1.

Bloom syndrome (BS) cells had an average replicative capacity (22.5 ± 2.8 PDs, and 32.8 ± 3.2 PDs in the presence of SB203580, n = 3 strains) that is not significantly different to NDFs (Table 1). In addition, the average experimental replicative capacity extension with SB203580 treatment and their growth rates were not significantly higher than seen for NDFs (Table 1; Fig. 1).

A single strain of Cockayne Syndrome A (CSA) cells was analysed. CSA(GM01856) cells achieved a replicative capacity of 21.1 PDs that was extended to 29.9 PDs with SB203580 treatment (Table 1). The growth rate also increased from 0.37 to 0.49 PDs/day (Table 1). Although the replicative capacity for control-treated CSA cells was significantly shorter than for normal cells (*p* < 0.045; *z*-test), it should be noted these cells had already been passaged seven times before analysis. The growth rates and experimental increase in replicative capacity with SB203580 were not statistically significant from NDFs (Table 1; Fig. 1).

Two strains of Cockayne Syndrome B (CSB) cells were analysed. Each had an average replicative capacity not significantly different from that seen in normal cells (Table 1). The replicative capacity of CSB cells in the presence of SB203580, however, was significantly greater than seen in SB203580-treated NDFs (*p* < 0.02; Table 1), although the replicative capacity extension was not (Fig. 1). The growth rates of both strains in the presence or absence of SB203580 was significantly greater than seen for NDFs (Table 1).

Three strains of Hutchinson-Gilford Progeria Syndrome (HGPS) cells were analysed. Their average replicative capacity was 14.3 ± 10.5 PDs, which is significantly shorter than seen for NDFs (*p* < 0.008). Average replicative capacity in the presence of SB202580 (19.5 ± 13.3 PDs) was also significantly reduced compared to SB203580-treated normal cells (*p* < 0.011). The percentage increase in replicative capacity was statistically different from that seen in NDFs at 46.1 ± 2.1 % (*p* < 0.043; Fig. 1).

A single strain of Seckel Syndrome (SS) cells, SS(GM09812), was analysed. It does not have a mutation in ATR (Alderton et al. 2004), and its aetiology is unknown. This strain had a replicative capacity in the presence or absence of SB203580 that was within the normal range (Table 1). The growth rate of SS(GM09812) cells, however, was statistically higher than that seen for NDFs.

Two strains of Nijmegen Breakage Syndrome (NBS) cells were studied. Both were diagnosed by clinical means (Der Kaloustian et al. 1996; Yamazaki et al. 1998) and no mutation data are available. Both strains had replicative capacities and growth rates not statistically different to that of NDFs (Table 1).

A single Rothmund Thomson (RTS) strain was studied. This had growth parameters within the range seen for NDFs, with the exception of the replicative capacity extension seen with SB203580, which was higher (Table 1). However, when the current data is added to previous results (Davis et al. 2012) fibroblasts from RTS had growth parameters not significantly different from NDFs (Table 1; Fig. 1).

Two strains of X-linked Dyskeratosis congenita syndrome cells were used, one of which [DKC(AG04645)] failed to proliferate sufficiently to allow growth data to be obtained. The remaining strain [DKC(GM01774)] managed 11.9 PDs that was extended to 16.5 PDs using SB203580 (Table 1), an experimental replicative capacity extension of 78 % taking into account the six PDs achieved prior to obtaining the cells. Its replicative capacity was significantly less than NDFs and showed a statistically significantly greater replicative capacity extension following SB203580 treatment (Table 1; Fig. 1). Its growth rate, although slightly below the range for normal cells, was not significantly different from normal NDFs using a *z*-test.

### Comparison of cellular morphologies of normal and progeroid syndrome cells

**Fig. 2:**
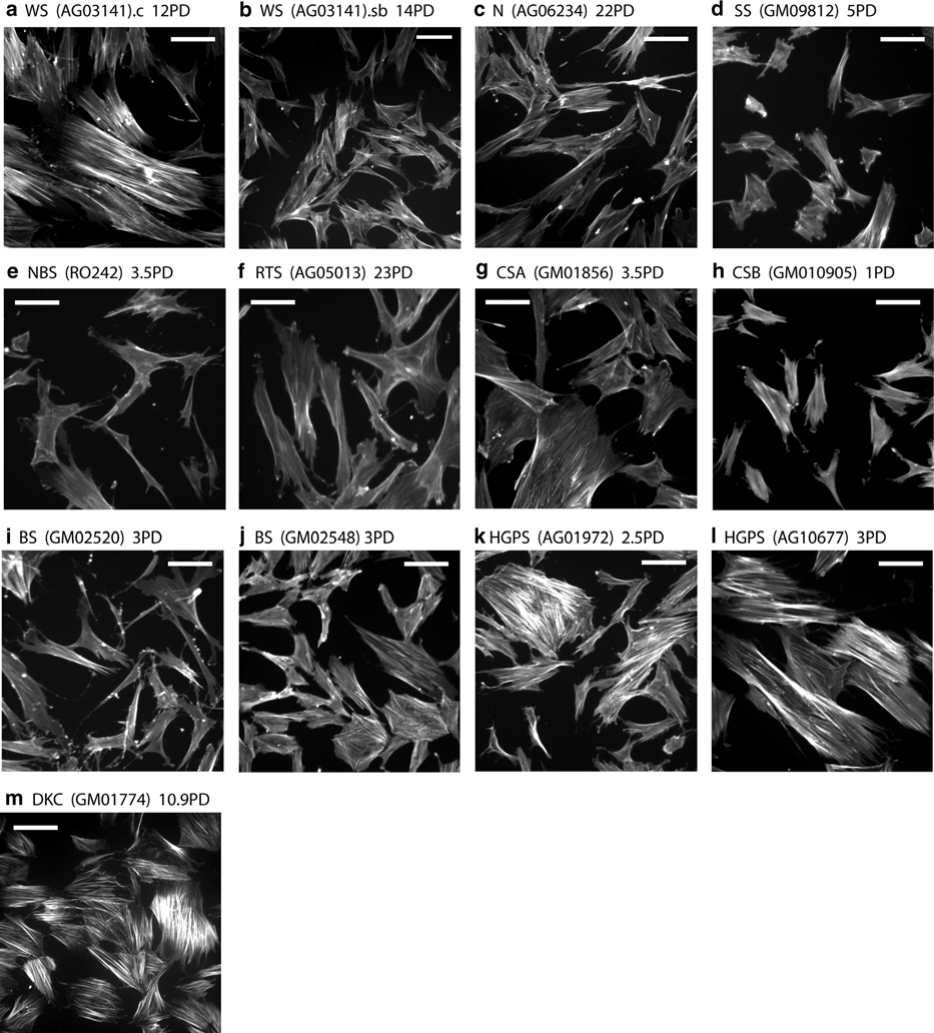
Observation of cellular morphology and F-actin stress fibres in progeroid and genomic instability syndrome fibroblasts. Cells were fixed and stained with phalloidin-FITC. For each panel, the cell type and PD values are given. For **a** and **b** a suffix.c are control cells and a suffix.sb are cells treated with SB203580: for the other syndromes the effects of SB203580 are not shown as the effects are small. For syndromes where the cellular morphology differs little from normal cells only representative strains are shown. *Bars* are 100 μm

A striking feature of young WS cells in culture is that the majority have an enlarged morphology and an extensive network of F-actin stress fibres (Davis et al. 2005) that can be visualised with phalloidin-FITC (Fig. 2a). Treatment of WS cells with SB203580 restores a normal morphology (Fig. 2b: compare with Fig. 2c). In contrast, cultures of normal fibroblasts show very few (<5 %) cells with extensive F-actin stress fibres (Fig. 2c), with the majority having a normal morphology (Davis and Kipling 2009). The progeroid and genomic instability syndromes used in this study can be divided into three distinct groups with respect to the cellular morphology of fibroblasts and its response to SB203580 treatment (Table 2).

**Table 2:** Summation of cellular morphologies and activation levels of p38 and HSP27

Cell strain	% Enlarged cells control^a^	% Enlarged cells SB203580^a^	p38-activation^b^	p-HSP27^b^
Normal				
N(AG16409)	<5	<5	±	+
Group 1 progeroid strain				
NBS(RO202)	<	<5	–	+
NBS(RO242)	<5	<5	–	+
RTS(GM05013)	<5	<5	±	+
SS(GM09812)	<5	<5	±	+
Group 2 progeroid strains				
BS(GM02520)	<5	<5	++++	+
BS(GM02932)	<15	<10	±	++
BS(GM02548)	<15	<10	±	++
CSA(GM01856)	<20	<20	++	++++
CSB(GM10903)	<15	<5	+	+++
CSB(GM10905)	<5	<5	–	++
HGPS(GM11498)	<5	<5	–	+
HGPS(GM01972)	<15	<10	–	+
HGPS(GM10677)	>80	>80	nd	nd
Group 3 progeroid strains				
DKC(GM01774)	>90	>90	nd	nd

^a^The proportion of enlarged cells is estimated from three fields within dish each containing >30 cells for control cells or SB203580 treated cells. Enlargement is a subjective measurement and is not quantitatively measured

^b^Based upon Fig. 3: −, absent; ±, barely detected or absent; +, detectable; ++, moderate, +++, strong; ++++, very high; *nd* not determined, all values are arbitrary based upon comparison between samples

Group 1 syndromes are those where the fibroblast morphology differs little from NDFs. The cultures having a few enlarged cells with F-actin stress fibres, but the majority having a normal morphology; these cultures are not affected by SB203580 treatment. This pattern is seen for both NBS strains, RTS(GM05013), and SS(GM09812) (Fig. 2d–f), and is similar to what has been reported previously in Ataxia-telangiectasia cells (Davis and Kipling 2009).

Group 2 syndromes are those that show inter-strain differences in fibroblast morphology and include the BS, HGPS, CSA and CSB strains. CSA(GM01856) cultures have up to 20 % cells with an enlarged morphology, although many of these enlarged cells do not have F-actin stress fibres (Fig. 2g) and their morphology was not affected by treatment with SB203580 (not shown). The morphology of the two CSB strains differs; CSB(GM10903) has up to 15 % of cells with an enlarged morphology with F-actin stress fibres that were ameliorated with SB203580 (not shown), whereas CSB(GM10905) (Fig. 2h) more closely resembles NDFs. The morphology of cells from BS varies between strains, with BS(GM02520) (Fig. 2i) closely resembling NDFs, whereas BS(GM02548) (Fig. 2j) and BS(GM02932) (not shown) have up to 15 % of the cells with an enlarged morphology with F-actin stress fibres. In the latter two BS strains treatment with SB203580 results in cultures with fewer cells with an enlarged morphology (not shown). A similar pattern is seen in two of the HGPS strains, with GM11498(HGPS) resembling NDFs (not shown) and HGPS(AG01972) (Fig. 2k) somewhat resembling the BS(GM02548) strain with SB203580 having a similar effect (not shown). In the third HGPS strain [HGPS(AG10677)] the majority of cells are enlarged with extensive F-actin stress fibres (Fig. 2l) and SB203580 has little effect on this (not shown). HGPS(AG10677) cells have a very short replicative capacity (3.8 PDs, Table 1) and were therefore very close to replicative senescence when analysed; it has been shown that senescent cells have an enlarged morphology that is not affected by SB203580 (Davis et al. 2005).

Group 3 strains are represented by the single DKC strain, DKC(GM01774). The cultures of this strain consisted almost exclusively of enlarged cells with extensive F-actin stress fibres (Fig. 2m) that were not affected by SB203580 treatment. Again these cells were close to replicative senescence by the time of analysis. For the second strain DKC strain (AG04645) insufficient cells were obtained for any analysis.

Although we have highlighted the differences, overall our data reveal only small differences in the fibroblast morphologies from the various syndromes and significant inter-strain similarities, particularly when compared to the reference WS strain (Fig. 2a). This may not be too surprising as the protein defects present in the various strains within each syndrome are very similar despite the different genetic lesions (see Supplementary Tables 1 and 2). The exception is HGPS where two strains result from the expression of progerin, and the other, HGPS(AG10677), is due to a mis-sense lamin A/C protein (Supplementary Table 1). Whilst it is possible that the differences seen in this latter strain result from the different proteins, it appears more likely that the differences result from the observation that HGPS(AG10677) was close to senescence when the study began.

### Characterisation of stress kinase effector pathways

**Fig. 3:**
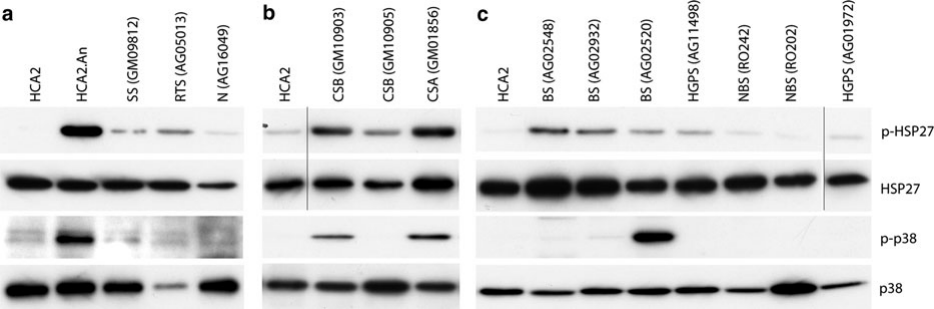
Immunoblot analysis of stress signalling proteins. Protein lysates were prepared from primary cells and from hTERT-immortalized HCA2 cells. Expression levels were compared for phosphorylated p38 (p-p38), p38, phosphorylated HSP27 (p-HSP27) and HSP27. HCA2^tert^ cells and HCA2^tert^ cells treated with anisomycin were used as negative and positive controls respectively for antibody efficacy (shown in **a**). Three sets of immunoblots were done and are shown as **a**, **b**, and **c**, for each immunoblot HCA2 cells were used as an internal standard (note that the *black lines* in **b** and **c** indicate that the single lanes have been cut and pasted from the same gels as the rest of the samples for each panel, however, the images have been handled in the same manner otherwise). Protein levels were normalised with respect to total p38

To assess the activation status of p38, proteins were extracted from young fibroblasts from each syndrome [with the exception of HGPS(AG10677) and the two DKC strains, where there were insufficient cells for protein analysis] and immunoblots were probed with antibodies for p38 and its activating phosphorylation. As a positive control anisomycin-treated HCA2 normal fibroblasts were used (Fig. 3). These data are summarised in Table 2.

Very low levels of phosphorylated p38 are found in N(AG16409), RTS(AG05013), SS(GM09812), BS(GM02548), BS(GM02932), HGPS(AG11498), HGPS(AG01972), NBS(RO202), NBS(RO242) and CSB(GM10905). Moderate levels are seen in the CSB(GM10903) and CSA(GM01856) strains. A high level of activated p38 is seen in BS(GM02520), despite low levels of phosphorylated HSP27 (Fig. 3).

The p38 target MK2 phosphorylates HSP27 on serines 78 and 82 (Huot et al. 1997). HSP27 phosphorylation is also related to the production of F-actin stress fibres. Low levels of phosphorylated HSP27 (p-HSP27) were observed in N(AG16409), RTS(AG05013), SS(GM09812), BS(GM02520), HGPS(AG11498), HGPS(AG01972) and the two NBS strains (Fig. 3). This is in agreement with the lack of enlarged cellular morphology and F-actin stress fibres seen in most of these strains. BS(GM02932) and BS(GM02548) show low to moderate levels of p-HSP27 (Fig. 3) and moderate levels of F-actin fibres compared to BS(GM02520). CSB(GM10903) has moderate levels of p-HSP27 and CSB(GM10905) has low p-HSP27 levels. The CSA(GM01856) strain has moderate to high p-HSP27 levels. In general, the level of p-HSP27 in the cell strains corresponds with the observation of F-actin stress fibres and corresponds to the activation status of p38, with the exception of BS(GM02520) where a high level of activated p38 does not produce a high level of p-HSP27 or F-actin stress fibres (Table 2).

### hTERT immortalisation of fibroblasts

Fibroblast strains from BS, SS, NBS, CSA, CSB, HGPS and the normal AG16409 (Table 3) were transduced with an amphotropic retrovirus expressing the catalytic subunit of telomerase, hTERT (see "Materials and Methods"). In each case, the resulting puromycin-resistant populations of cells were now telomerase positive as judged by the TRAP assay (Supplementary Figure 2) with the exception of NBS(RO242) for which a TRAP positive has not been possible to obtain. The replicative capacity of each strain was recorded. In every case telomerase conferred an extension of cellular replicative capacity beyond senescence, and all telomerase-transduced cells continue to proliferate at the time of writing.

**Table 3:** Growth parameters of progeroid fibroblasts infected with hTERT

Strain	PD at infection^a^	PD of control^b^	PDs since infection^c^	TRAP	Proliferating^d^
N(AG16409)	4	53	99	+	Y
HGPS(AG11498)	10	24	48	+	Y
BS(GM02548)	1	24	98	+	Y
CSA(GM01856)	1	22	101	+	Y
CSB(GM10903)	45	53	38	+	Y
SS(GM09812)	7	32	60	+	Y
NBS(RO242)	53	54	57	−	Y

^a^Replicative age of the culture when infection with hTERT retrovirus took place

^b^Replicative capacity of uninfected control cultures

^c^Number of PDs the terted strains had achieved post infection

^d^Culture is still proliferating at time of manuscript submission

As an example of normal cells, N(AG16409)^tert^ cells proliferated more than 46 PDs beyond senescence and are still proliferating (Table 3). Many different normal fibroblast strains have been immortalised since this was first achieved (Bodnar et al. 1998).

Fibroblasts from several of the syndromes used in this work have been immortalised previously, albeit using different strains. For HGPS and BS previous telomerase-reconstitution experiments have mostly used strains with a replicative capacity >35 PDs (Ouellette et al. 2000; Wallis et al. 2004), although the low replicative capacity strain HGPS(AG01972) has also been successfully immortalised with telomerase (Kudlow et al. 2008). To extend these studies we introduced hTERT into HGPS(AG11498) and BS(GM02548), both of which are low replicative capacity strains. Both HGPS(AG11498)^tert^ and BS(GM02548)^tert^ cells continued proliferation beyond senescence (Table 3). Thus, hTERT expression can successfully immortalise primary HGPS and BS strains irrespective of replicative capability.

The remaining progeroid and genomic instability syndromes used in this work have not previously been immortalised using hTERT. The CSA(GM01856)^tert^, CSB(GM10903)^tert^ NBS(RO242)^tert^, SS(GM09812)^tert^ and cells have all managed at least double the replicative capacity of uninfected controls and are still proliferating (Table 3).

**Fig. 4:**
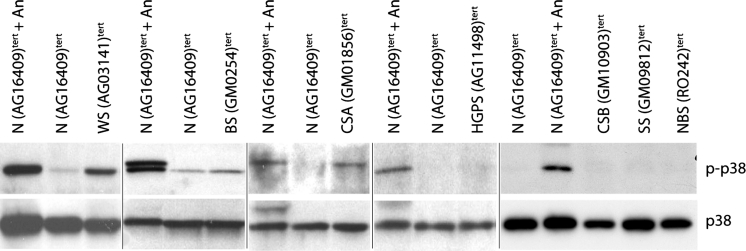
Assessment of stress p38 activity in TERT-transduced lines. Immunoblot for phosphorylated p38 (p-p38) and p38 [N(AG16409)^tert^ cells ± anisomycin treatment were used as controls]

The morphologies and stress fibre phenotypes of TERT-transduced fibroblasts from these syndromes were essentially the same as seen for the corresponding (uninfected) primary cells for each strain. The majority of the cells were small with few F-actin stress fibres visible (not shown). The level of p38 activity (as judged by p-p38) in each TERT-expressing line was similar to that seen for the corresponding primary fibroblasts. That is, if p38 is activated in the primaries it is also activated in the TERT-expressing samples; the exception being CSB(GM10903)^tert^ where p38 activity seems to have been suppressed (Fig. 4). These data suggest that telomerase-mediated immortalisation of these cell strains has not significantly perturbed this aspect of the phenotype.

## Discussion

Progeroid syndromes are human genetic disorders that show many, but not all, of the clinical characteristics of normal ageing (Martin et al. 1999; Hofer et al. 2005; Kudlow et al. 2008). They are widely used as model systems to study normal human ageing processes because, in those aspects where premature ageing occurs, the process and pathology are remarkably similar to that seen in normally aged individuals (Martin et al. 1999). In several of these syndromes, notably WS, HGPS and AT, premature in vivo ageing is associated with premature cellular ageing in vitro (Tollefsbol and Cohen 1984; Tchirkov and Lansdorp 2003; Bridger and Kill 2004). In addition, the premature senescence seen in WS fibroblasts is prevented using inhibitors of the stress-associated p38 MAPK (Davis et al. 2005).

Based upon these studies it has been postulated that premature cellular senescence may underlie many of the ageing features of these syndromes (Ostler et al. 2002; Faragher et al. 2009). This in turn raises the question, is shortened cellular replicative capacity a general characteristic of progeroid syndromes? In this study we have therefore determined the growth characteristics and replicative capability of fibroblasts from several progeroid syndromes and investigated the role, if any, played by p38 MAP kinase.

The HGPS strains used in this work have a reduced replicative capacity that is only slightly extended by SB203580 treatment, but they do not have an “aged” morphology [with the exception of HGPS(AG10677), which was close to replicative senescence at the start of this work] and no activated p38. HGPS individuals have a high rate of ageing and a much reduced lifespan, and the syndrome is associated with inflammatory conditions such as atherosclerosis and osteoporosis. Thus, the HGPS cells used in this study have a reduced replicative capacity, but one that is not corrected by SB203580.

Cockayne Syndrome has two variants (CSA and CSB), and it is clear from this work that CSB fibroblasts do not have an obvious replicative capacity defect, whereas CSA fibroblasts may do so (Thompson and Holliday 1983). Both CSA and CSB(GM10903) fibroblasts show moderate levels of cells with a stressed morphology and activated p38; however, SB203580 does not significantly extend the proliferative capacity of CS fibroblasts beyond that seen in normals. In addition, SB203580 does not ameliorate the enlarged morphology seen in the CSA fibroblasts, although it does so in CSB fibroblasts (Table 2). It is possible that the enlarged CSA cells have undergone senescence in a p38-independent fashion, since it has been shown that SB203580 has no effect of the morphology of cells that have undergone telomere-dependent senescence (Davis et al. 2005). The presence of phosphorylated p38 and HSP27 support this, because both are activated upon telomere-dependent replicative senescence (Iwasa et al. 2003; Davis et al. 2005). With CSB(GM10903) cells, the reduction in the level of cells with an altered cellular morphology using SB203580, and the observation of activated p38 and phosphorylated HSP27, suggests the presence of a low level of SIPS. CS individuals have characteristic facies, thin hair, cachexia, retinal degeneration, hearing loss, neurodegeneration (cerebellar ataxia), and cataracts (Kraemer et al. 2007). Their clinical course typifies premature ageing and usually results in early death, with CSA individuals having a shortened lifespan (death occurring in the second or third decade), and CSB individuals dying in the first decade (Rapin et al. 2000).

BS fibroblasts have a replicative capacity towards the lower end of the normal range (Thompson and Holliday 1983; Ouellette et al. 2000), but do not show an aged morphology (although small numbers of enlarged cells are present in two strains), and p38 is only activated in a single strain. BS individuals have a moderately reduced lifespan (Hofer et al. 2005); however, although classified as a progeroid syndrome, there is little evidence of a premature ageing defect apart from a high incidence of type II diabetes in young BS individuals, and an elevated cancer incidence.

RTS Syndrome fibroblasts do not show a replicative defect and little by way of an aged morphology, and RTS individuals have a normal lifespan (Hofer et al. 2005). However, RTS individuals do show moderate ageing characteristics, including alopecia, gray hair, cataracts, and poikiloderma. p38 is not activated in the one RTS fibroblast strain used in this study.

With NBS, the two strains of cells used here have no replicative defect, no aged-cell morphology, and no activated p38. In addition, p38 inhibition has only a small effect on fibroblast growth. The progeroid features described for NBS, such as sparse hair and distinctive ‘bird-like’ facies, do increase with age, but are relatively mild and there are few inflammatory features (Seemanova et al. 1985). However, the data on NBS are potentially confounded by many individuals dying at a young age as a result of cancer.

Fibroblasts from Seckel Syndrome do not have a reduced replicative capacity and no p38 activation. SS individuals show moderate ageing with few inflammatory features (O’Driscoll et al. 2004), although accelerated ageing is clearly present in the SS mouse model (Murga et al. 2009).

The X-linked DKC fibroblasts used in this study are known to have a shortened replicative capacity but this is due to deficiencies in telomere maintenance (Wong and Collins 2006). Whether there is an additional component due to stress-induced senescence is not clear from our study for technical reasons, there being insufficient DKC cells for detailed analysis. The ageing characteristics in DKC are many, such as alopecia, gray hair, osteoporosis and abnormal skin pigmentation (Dokal 2000), but resemble those seen in telomerase deficient mice (Rudolph et al. 1999) and thus may result from telomere dysfunction-induced cellular senescence.

Thus, although fibroblasts from several of the progeroid syndromes show premature cellular senescence, there appears to be no clear relationship between replicative cellular capacity of the cell strains used and the presence of premature ageing features. However, it may be that the presence of accelerated cellular senescence correlates with the severity of the ageing phenotype, e.g., in WS, HGPS, and DKC and AT, the ageing features are marked and the lifespan of individuals is reduced. However, with RTS and CS the ageing features are also marked, but fibroblasts have a normal replicative capacity. Alternatively, tissue specificity may play a role, since dermal fibroblasts from WS, HGPS, DKC and AT all show premature senescence, and a notable feature of these particular progeroid syndromes is skin ageing. However, this observed lack of accelerated ageing of fibroblasts (or SIPS) in no way suggests that features of the ageing seen in progeroid syndromes are not due to replicative cellular senescence, or the presence of senescent cells.

In addition, with the exception of WS, there appears to be no role for the stress kinase p38 in the premature senescence seen in fibroblasts from the progeroid syndromes used in this study. With regards to p38, there also appears to be no correlation between p38 activation and the presence of inflammatory features. Thus it would be useful to examine other cell types from these syndromes, e.g., vascular smooth muscle cells in HGPS are reported to be depleted from the large arteries in HGPS individuals (Stehbens et al. 2001), which may be due to accelerated senescence or apoptosis. Overall, our study demonstrates that accelerated fibroblast senescence and p38 activation are features of some, but not all, genome instability and progeroid syndromes.

## Electronic supplementary material

Below is the link to the electronic supplementary material.

Supplementary material 1 (DOC 93 kb)

Supplementary material 2 (DOC 34 kb)

Supplementary material 3 (TIFF 4810 kb)

Supplementary material 4 (TIFF 840 kb)

Supplementary material 5 (DOC 27 kb)
