# Supplementary material for: p38 MAPK stress signalling in replicative senescence in fibroblasts from progeroid and genomic instability syndromes
Source: Biogerontology. 2012 Oct 31;14(1):47–62. doi: 10.1007/s10522-012-9407-2 (PMC3627027; doi:10.1007/s10522-012-9407-2)
Supplement: Supplementary file 1 — Supplementary material 1 (DOC 93 kb) [file 10522_2012_9407_MOESM1_ESM.doc]

**p38 MAPK stress signalling in replicative senescence in fibroblasts from progeroid and**

**genomic instability syndromes**

**Biogerontology**

**H. Tivey, A. Brook, M. Rokicki, D. Kipling, T. Davis**

**Cardiff University School of Medicine, UK, davist2@cardiff.ac.uk**

**Supplementary Table 1** Cell strains used in this work

Strain ^a^ Replicative ^b^ Gene ^c^ Protein ^c^ Age of Reference

History affected expression donor

**Wild-type (N)**

N(AG04552) 10 PDs n/a n/a 65 Coriell Data sheet

N(AG06234) 15 PDs n/a n/a 17 Coriell Data sheet

N(AG09603) 16 PDs n/a n/a 82 Coriell Data sheet

N(AG11020) 9 PDs n/a n/a 78 Coriell Data sheet

N(AG11081) 13 PDs n/a n/a 78 Coriell Data sheet

N(AG13152) 6 PDs n/a n/a 79 Coriell Data sheet

N(AG13156) 7 PDs n/a n/a 44 Coriell Data sheet

N(AG16409) 4 PDs n/a n/a 14 Coriell Data sheet

**Bloom Syndrome (BS)**

BS(GM02548) p8 *BLM* protein absent 6 (Barefield, 2012)

BS(GM02520) p11 *BLM* protein absent 10 Coriell Data sheet

BS(GM02932) p6 *BLM* protein absent 28 (Killen et al. 2009)

**Cockayne Syndrome type A (CSA)**

CSA(GM01856) p7 *ERCC8* Reduced level 13 (Ridley et al. 2005)

missence protein

**Cockayne Syndrome type B (CSB)**

CSB(GM10903) p1 *ERCC6* protein absent 9 (Colella et al. 2000)

CSB(GM10905) p1 *ERCC6* protein absent 10 (Colella et al. 2000)

**Hutchinson-Gilford progeria syndrome (HGPS)**

HGPS(AG01972) p10 *LMNA* progerin present 14 (Eriksson et al. 2003)

HGPS(AG10677) p10 *LMNA* missence protein 4 (Bridger & Kill 2004)

HGPS(AG11498) 10 PDs *LMNA* progerin present 14 (Eriksson et al. 2003)

**Seckel Syndrome** **(SS)** **^d^**

SS(GM09812) p4 NK NK 15 (Alderton et al. 2004)

**Nijmegen Breakage Syndrome (NBS)**

NBS(RO202) p1 NK NK NK clinical diagnosis ^e^

NBS(RO242) p1 NK NK NK clinical diagnosis ^e^

**X-linked Dyskeratosis congenita (DKC)**

DKC(GM01774) 6PD *DKC1* missence prot at 7 (Wong & Collins 2006)

WT levels (Mitchell et al. 1999)

DKC(AG04645) 4PD *DKC1* missence prot at 11 (Wong & Collins 2006)

WT levels (Mitchell et al. 1999)

**Rothmund Thomson Syndrome (RTS)**

RTS(AG05013) 21 PDs *RECQL4* Protein absent 10 (Yin et al. 2004)

RTS(AG17524) p9 *RECQL4* NK 4 Coriell Data sheet

RTS(AG18371) p1 *RECQL4* Protein absent 12 (Petkovic et al. 2005)

RTS(AG18375) p1 *RECQL4* NK 22 Coriell Data sheet

**Ataxia Telangiectasia (AT)**

AT(AG03058) 12 PDs *ATM* protein absent 14 (Heinloth et al. 2003)

AT(AG04405) 9 PDs *ATM* protein absent 6 (de Toledo et al. 2000)

AT(GM05823) p15 *ATM* protein absent 18 (Tang et al. 2002)

**Werner Sysndrome (WS)**

WS(AG03141) 9 PDs *WRN* protein absent 30 (Nyunoya et al. 2009)

WS(AG05229) 7 PDs *WRN*  protein absent 25 (Marciniak et al. 1998)

WS(AG12795) 11 PDs *WRN* NK 19 clinical diagnosis

^a^ To avoid confusion when referring to cell strains in this paper a prefix has been added to the strain code.

^b^ Number of population doublings (PDs) or passages (p) achieved at Coriell Repository prior to receipt of cells.

^c^ n/a = not applicable; NK = not known.

^d^ GM09812 is not mutated for ATR (Alderton et al. 2004, Stokes et al. 2007).

^e^ strains obtained from W. J. Kleijer and described as variants of Nijmegen Breakage syndrome (Der Kaloustian et al. 1996, Yamazaki et al. 1998).

**References**

Alderton GK, Joenje H, Varon R, Borglum AD, Jeggo PA, O'Driscoll M (2004) Seckel syndrome exhibits cellular features demonstrating defects in the ATR-signalling pathway. Hum Mol Genet 13:3127-3138

Barefield C, Karlseder J (2012) The BLM helicase contributes to telomere maintenance through processing of late-replicating intermediate structures. Nucleic Acids Res 40:7358-7367

Bridger JM, Kill IR (2004) Aging of Hutchinson-Gilford progeria syndrome fibroblasts is characterised by hyperproliferation and increased apoptosis. Exp Gerontol 39:717-724

Colella S, Nardo T, Botta E, Lehmann AR, Stefanini M (2000) Identical mutations in the CSB gene associated with either Cockayne syndrome or the DeSanctis-cacchione variant of xeroderma pigmentosum. Hum Mol Genet 9:1171-1175

de Toledo SM, Azzam EI, Dahlberg WK, Gooding TB, Little JB (2000) ATM complexes with HDM2 and promotes its rapid phosphorylation in a p53-independent manner in normal and tumor human cells exposed to ionizing radiation. Oncogene 19:6185-6193

Der Kaloustian VM, Kleijer W, Booth A, Auerbach AD, Mazer B, Elliott AM, Abish S, Usher R, Watters G, Vekemans M, Eydoux P (1996) Possible new variant of Nijmegen breakage syndrome. Am J Med Genet 65:21-26

Eriksson M, Brown WT, Gordon LB, Glynn MW, Singer J, Scott L, Erdos MR, Robbins CM, Moses TY, Berglund P, Dutra A, Pak E, Durkin S, Csoka AB, Boehnke M, Glover TW, Collins FS (2003) Recurrent de novo point mutations in lamin A cause Hutchinson-Gilford progeria syndrome. Nature 423:293-298

Heinloth AN, Shackelford RE, Innes CL, Bennett L, Li L, Amin RP, Sieber SO, Flores KG, Bushel PR, Paules RS (2003) ATM-dependent and -independent gene expression changes in response to oxidative stress, gamma irradiation, and UV irradiation. Radiat Res 160:273-290

Killen MW, Stults DM, Adachi N, Hanakahi L, Pierce AJ (2009) Loss of Bloom syndrome protein destabilizes human gene cluster architecture. Hum Mol Genet 18:3417-3428

Marciniak RA, Lombard DB, Johnson FB, Guarente L (1998) Nucleolar localization of the Werner syndrome protein in human cells. Proc Natl Acad Sci U S A 95:6887-6892

Mitchell JR, Wood E, Collins K (1999) A telomerase component is defective in the human disease dyskeratosis congenita. Nature 402:551-555

Nyunoya T, Monick MM, Klingelhutz AL, Glaser H, Cagley JR, Brown CO, Matsumoto E, Aykin-Burns N, Spitz DR, Oshima J, Hunninghake GW (2009) Cigarette smoke induces cellular senescence via Werner's syndrome protein down-regulation. Am J Respir Crit Care Med 179:279-287

Petkovic M, Dietschy T, Freire R, Jiao R, Stagljar I (2005) The human Rothmund-Thomson syndrome gene product, RECQL4, localizes to distinct nuclear foci that coincide with proteins involved in the maintenance of genome stability. J Cell Sci 118:4261-4269

Ridley AJ, Colley J, Wynford-Thomas D, Jones CJ (2005) Characterisation of novel mutations in Cockayne syndrome type A and xeroderma pigmentosum group C subjects. J Hum Genet 50:151-154

Stokes MP, Rush J, Macneill J, Ren JM, Sprott K, Nardone J, Yang V, Beausoleil SA, Gygi SP, Livingstone M, Zhang H, Polakiewicz RD, Comb MJ (2007) Profiling of UV-induced ATM/ATR signaling pathways. Proc Natl Acad Sci U S A 104:19855-19860

Tang D, Wu D, Hirao A, Lahti JM, Liu L, Mazza B, Kidd VJ, Mak TW, Ingram AJ (2002) ERK activation mediates cell cycle arrest and apoptosis after DNA damage independently of p53. J Biol Chem 277:12710-12717

Wong JM, Collins K (2006) Telomerase RNA level limits telomere maintenance in X-linked dyskeratosis congenita. Genes Dev 20:2848-2858

Yamazaki V, Wegner RD, Kirchgessner CU (1998) Characterization of cell cycle checkpoint responses after ionizing radiation in Nijmegen breakage syndrome cells. Cancer Res 58:2316-2322

Yin J, Kwon YT, Varshavsky A, Wang W (2004) RECQL4, mutated in the Rothmund-Thomson and RAPADILINO syndromes, interacts with ubiquitin ligases UBR1 and UBR2 of the N-end rule pathway. Hum Mol Genet 13:2421-2430
