# Supplementary material for: p38 MAPK stress signalling in replicative senescence in fibroblasts from progeroid and genomic instability syndromes
Source: Biogerontology. 2012 Oct 31;14(1):47–62. doi: 10.1007/s10522-012-9407-2 (PMC3627027; doi:10.1007/s10522-012-9407-2)
Supplement: Supplementary file 2 — Supplementary material 2 (DOC 34 kb) [file 10522_2012_9407_MOESM2_ESM.doc]

**p38 MAPK stress signalling in replicative senescence in fibroblasts from progeroid and**

**genomic instability syndromes**

**Biogerontology**

**H. Tivey, A. Brook, M. Rokicki, D. Kipling, T. Davis**

**Cardiff University School of Medicine, UK, davist2@cardiff.ac.uk**

**Supplementary Table 2** Genetic lesions in the strains used in this work

Strain ^a^ lesion

**Bloom Syndrome (BS)**

BS(GM02520) donor subject is a compound heterozygote: one allele has a deletion at nucleotide

2923 of the RECQL3 gene [2923delC] resulting in a frameshift [fs:974+23-X]

and a second allele has a deletion of exon 15 resulting in a frameshift [fs:941+26-X].

BS(GM02548) donor subject is a compound heterozygote: one allele has a 1-bp insertion at nucleotide 1544 of the RECQL3 gene [1544insA] resulting in a frameshift and premature termination [fs:514+1-X] and a second allele has a C>T transition at nucleotide 2328 of the RECQL3 gene [2328C>T] resulting in a substitution of a termination codon for glutamine at codon 752 [Gln752Ter (Q752X)].

BS(GM02932) donor subject is homozygous for a 6-bp deletion/7-bp insertion [6-bp del/7-bp ins] at nucleotide 2,281 of the open reading frame of the RECQL3 gene, which results in a frameshift and a stop codon

**Cockayne Syndrome type A (CSA)**

CSA(GM01856) compound heterozygote for a missense mutation (A160V) and a previously described nonsense mutation (E13X).

**Cockayne Syndrome type B (CSB)**

CSB(GM10903) donor subject is homozygous for a C>T transition at nucleotide 2282 (2282C>T) in the

ERCC6 gene, resulting in a nonsense mutation at codon 735 [ARG735TER (R735X)];

the donor subject is also homozygous for a silent change at nucleotide 2830 [a C>T

transition (2830C>T; GLY917GLY)].

CSB(GM10905) donor subject is homozygous for a C>T transition at nucleotide 2282 (2282C>T) in the ERCC6 gene, resulting in a nonsense mutation at codon 735 [ARG735TER (R735X)]; the donor subject is also homozygous for a silent change at nucleotide 2830 [a C>T transition (2830C>T; GLY917GLY)].

**Hutchinson-Gilford progeria syndrome (HGPS)**

HGPS(AG01972) de novo single base substitution, a C>T change at nucleotide 2036 (2036C>T), which

results in a silent change at codon 608 [Gly608Gly (G608G)] in exon 11 of the Lamin

A gene (LMNA)

HGPS(AG10677) Glu-to-Lys substitution at codon 145 [Glu145Lys (E145K)] in exon 2 of the Lamin A

gene (LMNA).

HGPS(AG11498) de novo single base substitution, a C>T change at nucleotide 2036 (2036C>T), which

results in a silent change at codon 608 [Gly608Gly (G608G)] in exon 11 of the Lamin

A gene (LMNA).

**X-linked Dyskeratosis congenita (DKC)**

DKC(GM01774) donor subject is hemizygous for an in frame 3 bp deletion of nucleotides 201_203 of the DKC1 gene (201_203delCTT) resulting in the deletion of leucine at position 37 [Leu37del]

DKC(AG04645) donor subject is hemizygous for an in frame 3 bp deletion of nucleotides 201_203 of the DKC1 gene (201_203delCTT) resulting in the deletion of leucine at position 37 [Leu37del]

**Rothmund Thomson Syndrome (RTS)**

RTS(AG05013) heterozygous for: g.2492del2, framshift, and 3’ splice site (kitao et al., 1999, Nature 22, p82).

**Werner Syndrome (WS)**

WS(AG03141) Homozygous for a C to T transition at nucleotide 2476 in the WRN gene (2476C>T), resulting in a stop codon at 748 {Gln748TER (Q748X)}.

^a^ Only for strains used in this study
