# Supplementary figures and images for: p38 MAPK stress signalling in replicative senescence in fibroblasts from progeroid and genomic instability syndromes
Source: Biogerontology. 2012 Oct 31;14(1):47–62. doi: 10.1007/s10522-012-9407-2 (PMC3627027; doi:10.1007/s10522-012-9407-2)

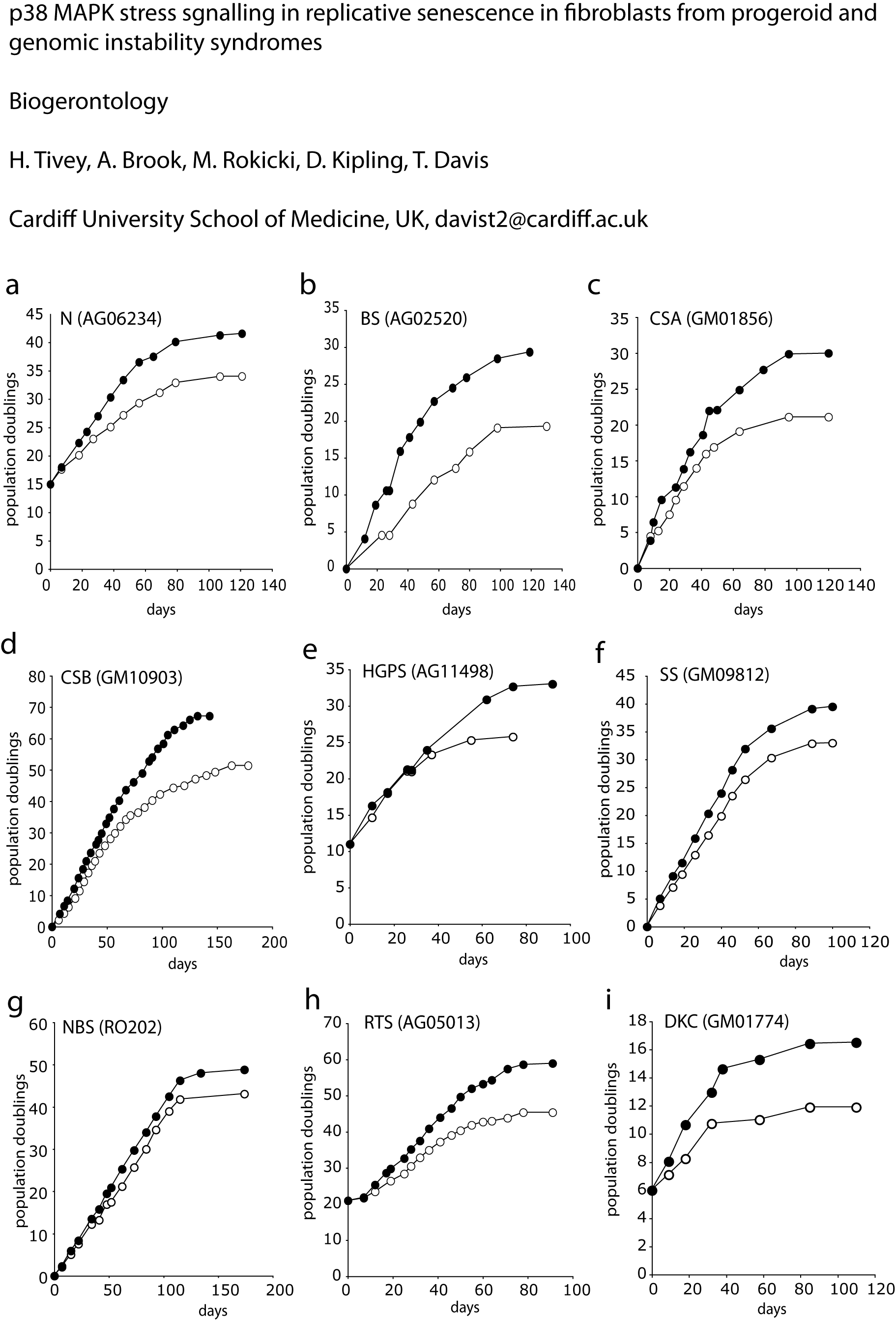

Supplement: Supplementary file 3 — Supplementary material 3 (TIFF 4810 kb) [file 10522_2012_9407_MOESM3_ESM.tif]

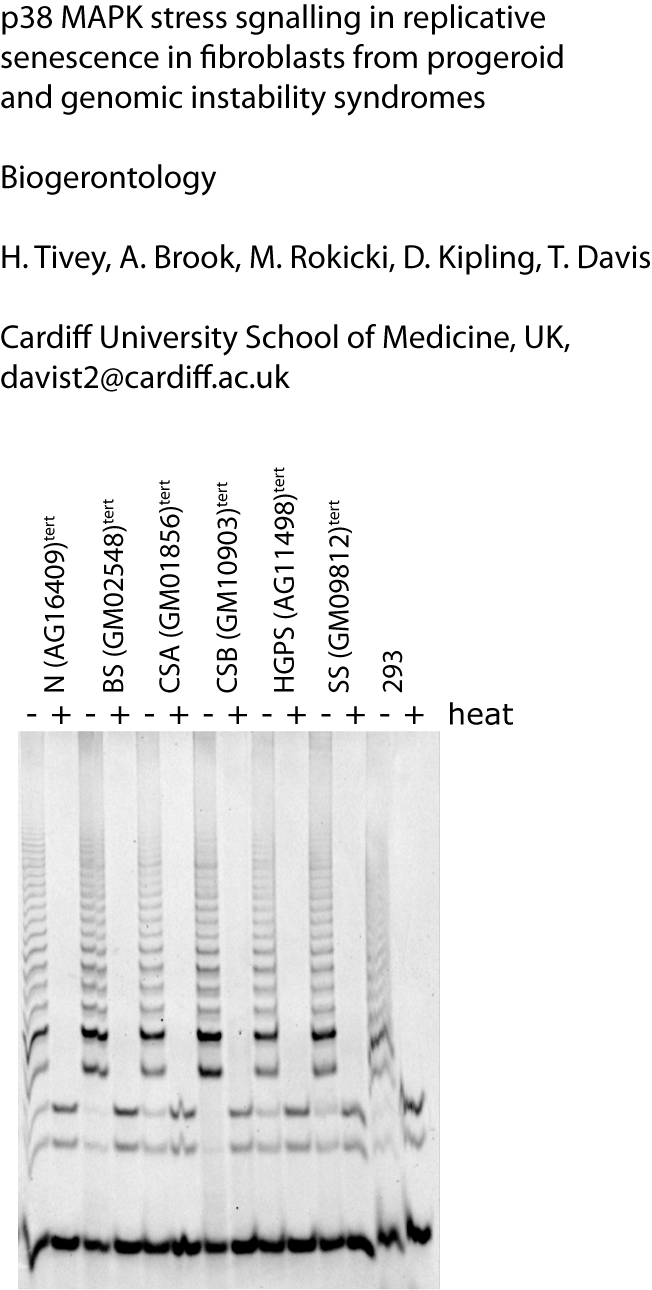

Supplement: Supplementary file 4 — Supplementary material 4 (TIFF 840 kb) [file 10522_2012_9407_MOESM4_ESM.tif]
