# Supplementary material for: p38 MAPK stress signalling in replicative senescence in fibroblasts from progeroid and genomic instability syndromes
Source: Biogerontology. 2012 Oct 31;14(1):47–62. doi: 10.1007/s10522-012-9407-2 (PMC3627027; doi:10.1007/s10522-012-9407-2)
Supplement: Supplementary file 5 — Supplementary material 5 (DOC 27 kb) [file 10522_2012_9407_MOESM5_ESM.doc]

**p38 MAPK stress signalling in replicative senescence in fibroblasts from progeroid and genomic instability syndromes**

**Biogerontology**

**H. Tivey, A. Brook, M. Rokicki, D. Kipling, T. Davis**

**Cardiff University School of Medicine, UK,** [**davist2@cardiff.ac.uk**](mailto:davist2@cardiff.ac.uk)

**Supplementary Figure legends**

**Supplementary Fig. 1** Growth of fibroblasts from progeroid and genomic instability syndromes with or without SB203580. (a-i) Fibroblasts were grown in standard EMEM with no supplementation (–○–), with continual daily supplementation with SB203580 (–●–). Growth measured as PDs versus days. Only representative growth curves for each syndrome are given.

**Supplementary Fig. 2** TRAP assay indicating telomerase activity in TERT-transduced lines. 293 cells are used as a positive control: samples were heat treated at 85^o^C for 10 mins (+) to destroy the telomerase activity.
